# The association between employment and family histories and caregiving in later life. European findings based on SHARE and ELSA

**DOI:** 10.1007/s10433-026-00919-8

**Published:** 2026-04-29

**Authors:** Christian Deindl, Giorgio Di Gessa, Morten Wahrendorf, Maike van Damme, Jeroen Spijker, Rebecca Lacey, Baowen Xue, Markus King, Anne McMunn

**Affiliations:** 1 Department of Social Sciences, Institute of Sociology, TU Dortmund University, Dortmund, Germany; 2 Epidemiology and Public Health, University College London, London, UK; 3 Institute of Medical Sociology, Medical Faculty and University Hospital Düsseldorf, Heinrich Heine University, Düsseldorf, Germany; 4 Center for Demographic Studies (CED), Bellaterra, Spain; 5 Institute for Advanced Family Studies, Universitat Internacional de Catalunya, Barcelona, Spain; 6 School of Health and Medical Sciences, University of London, City St. George’s, London, UK; 7 Department of Education, Faculty of Human Sciences, University of Potsdam, Potsdam, Germany

**Keywords:** Life course, Europe, Informal care, Sequence analysis

## Abstract

Caregiving can be time-consuming, and it can be challenging for caregivers to combine caregiving tasks with other obligations. While we know a lot about the problems of combining informal care with employment and family obligations, past employment and family histories are seldom discussed. This study tries to fill this gap by examining the association between employment and family histories and caregiving in later life. We used pooled data from the Survey of Health, Ageing and Retirement in Europe (SHARE, waves 1 and 2) and the English Longitudinal Study of Ageing (ELSA, waves 2 and 3) and combined them with the life history interviews from both surveys conducted in wave 3. First, we used sequence analysis and cluster analysis to analyze employment, partnership, and children histories between 25 and 50 years of age, separately for men and women. Then, we used logistic regression analysis to examine the relationships between these clusters and informal caregiving at ages 50 and above. Results indicate that women who stayed home (homemakers) and those who were self-employed were more likely to provide informal care in later life. Partnership histories matter only for men. Men who were separated were less involved in in-household caregiving and more in care provision outside of the household. Furthermore, childless men and women were more likely to be caregivers. Finally, differences in long-term care policies across countries significantly affected the likelihood of informal caregiving for men and women.

## Introduction

Care can be a time-demanding task. As a result, caregivers often face difficulties combining care responsibilities with other obligations. Several studies investigated the relationship between care and employment (Moussa 2019) and challenges in combining care for older people with other family duties (Grundy and Henretta 2006). Further, household tasks, childcare, and informal care remain unequally distributed between men and women (McMunn et al. 2020a, b), and difficulties in combining care responsibilities with employment, together with women’s weaker labor market attachment, help explain part of the gender gap for informal care provision (Bassoli and Brugiavini 2024; Scheuermann et al. 2024). Although we know much about combining informal care with employment and family responsibilities (Carmichael et al. 2010; Carmichael & Ercolani 2016; Rubin and White-Means 2009), employment and family histories are seldom considered (see (McMunn et al. 2020a, b) for an exception). This is surprising because later-life employment and family conditions often reflect earlier-life events; ignoring such path dependencies may bias estimates of their impact on caregiving later in life. In this article, we analyze the association between employment and family histories and informal care provided inside and outside the household. Using harmonized life history data from the Survey of Health, Age and Retirement in Europe (SHARE) and the English Longitudinal Study of Ageing (ELSA), we conduct the analysis based on large European datasets and additionally investigate the role of different care regimes in shaping informal care.

### Informal care

We define (informal) care as help or assistance—usually unpaid—provided to family members or friends with mental and physical health conditions, disabilities, and addictions. The most important reason to provide care is that someone close to you has health issues (Broese van Groenou and Boer 2016; Di Gessa and Deindl 2024). In most cases, potential caregivers feel obligated to care for someone they feel close to (Bengtson and Roberts 1991; Gans and Silverstein 2006) if they have the opportunities to do so (Brandt and Deindl 2013). These opportunities and restrictions (“perceived barriers” (Broese van Groenou and Boer 2016)) are important for taking on care responsibilities. Children, for example, who live far from their parents are less likely to take on care responsibilities than siblings who live nearby (Haberkern and Szydlik 2010). Additionally, socioeconomic resources play an important role. Typically, people with higher wealth, income, and education are less likely to care for a spouse or parents (Quashie et al. 2022), partly because of higher labor market involvement and the ability to afford assistance. Family composition and the wider social network (Ho et al. 2023; Spijker et al. 2022) are additional sources of support that determine informal care.

## Employment, family, and informal care

Employment and family responsibilities strongly shape the likelihood of taking on care responsibilities, as these domains compete for limited time, constraining the availability for caregiving (McMunn et al. 2020a). In many cases, caregiving leads to reduced working hours, especially for women (Moussa 2019). Sandwich caregivers—those also responsible for a dependent child—often report reducing working hours as well (Barker et al. 2017; Hammer & Neal 2008).

A life course approach can shed new light on these connections. Not only current employment matters: Earlier-life histories may leave an imprint on the life course that elevates the likelihood of taking on care responsibilities later in life. Employment and family histories typically follow certain patterns (Engels et al. 2019): Men are more often devoted to full-time employment, while a high percentage of women stay home and take over family responsibilities (Engels et al. 2021). Women also more often leave work or work part-time when taking on family responsibilities, such as childcare or informal care (McMunn et al. 2015; Schober 2013). This weaker attachment of women to employment across the life course might explain gender differences in care uptake later in life.

Beyond employment, family life also matters for care later in life by shaping exposure to potential care recipients and embedding individuals in kinship structures that carry expectations of support. Because having someone in need of care is a key determinant of caregiving, people who remain single throughout adulthood have fewer potential care recipients, whereas married persons have partners and parents-in-law who may need care. Yet, to date, only a few studies have considered care histories and care over the life course (Glaser et al. 2024; Moen et al. 1994; Raiber et al. 2022; Verbakel et al. 2023a, b), with even fewer combining employment and care histories (Carmichael and Ercolani 2016), although it is well known that conditions later in life reflect decisions and constraints earlier on (Crosnoe an Elder 2004; Elder et al. 2003; O'Rand and Henretta 1999). Carmichael and Ercolani (2016) used UK panel data spanning 15–20 years and combined employment and care histories. They found five employment-care clusters (full-time careers, evolving careers, part-time careers, caring intensive, and decaying careers) that appear “predictable and persistent” (Carmichael & Ercolani 2016: 10), suggesting that employment and care follow specific pathways over the life course. Moen et al. (1994) found that most women cared for a parent or spouse at some point, typically during late midlife. They also found that caregiving did not interfere with employment; rather, women were more likely to give up caregiving than employment. While both studies found gender differences, Carmichael and Ercolani (2016) additionally found that age and attitudes matter: Women, older people, and those with more traditional views were more likely to become caregivers.

To our knowledge, McMunn et al. (2020b) are the only study to consider the connection between employment and family history and caregiving. Using the 1958 National Child Development Study (NCDS), they found that the number of years spent married was positively correlated with both the likelihood and intensity of informal care. They also found that women’s part-time employment and men’s full-time employment were positively correlated with informal care.

Given this evidence, our first hypothesis is that individuals with a work-oriented life course are less likely to engage in informal care later in life, while those with a family-oriented life course (e.g., part-time employment, having children, and a life-long partner) are more likely to provide informal care later in life (ages 50 and above) (Hypothesis 1). A family-oriented life course implies both greater exposure to potential care recipients (a partner, parents-in-law, etc.) and greater familiarity with caregiving tasks.

Additionally, we expect strong gender differences in men’s and women’s life courses and in how employment and family histories shape caregiving. Our second hypothesis is that men with a traditional labor market trajectory and less family involvement are also less likely to be involved in informal care later in life if they have access to someone else (a partner) to take over their care responsibilities (Hypothesis 2).

Care location is an important distinction. Care can be provided inside or outside the caregiver’s household. Care inside the household is typically more intensive and most often provided to a spouse. Care outside the household can vary in intensity and is most often care for parents (in-laws) (Kaschowitz and Brandt 2017; Quashie et al. 2022). We therefore also expect life histories to differ by care location. Our third hypothesis is that people who spend most of their lives in a stable partnership are more likely to care both inside and outside the household (Hypothesis 3).

## Informal care and the welfare state

Context conditions, especially long-term care policies, are important when considering informal caregiving (Verbakel et al. 2023a). The impact of country conditions is often discussed in terms of crowding out versus crowding in. The idea that higher public transfers crowd out private, informal support has received much attention (Kohli 1999; Künemund and Rein 1999). However, most publications rather suggest a form of specialization: When the state assumes certain support duties, families provide not less but different types of support (Brandt 2013; Daatland and Herlofson 2003).

Other approaches group countries based on long-term care policies. For instance, Saraceno and Keck (2010) group countries by the generosity of public and financial support, distinguishing between “familialism-by-default” (little/no public support for formal care), “supported familialism” (some public support, mostly financial, for (private) care), and “defamilialism” (strong public support and a lower need for informal care). More recently, Van Damme et al. (2025) developed a regime typology for long-term care and found three regimes: (1) strong defamilialism/supported familialism, with both care in kind and financial aid by the state to reduce family care responsibilities, (2) moderate defamilialism/supported familialism, with a medium amount of formal elder-care support and limited care in kind, and (3) familialism-by-default, with little or no state support for family care, placing responsibility entirely on families. Our fourth hypothesis is that in countries with limited public support (familialism-by-default), care is mostly organized by families, whereas in countries with generous support for informal care (strong defamilialism), family involvement in care is less common (Hypothesis 4).

## Data and method

We used data from the Survey of Health, Age and Retirement in Europe (SHARE) (Börsch-Supan et al. 2013) and the English Longitudinal Study of Ageing (ELSA) (Steptoe et al. 2013). Both surveys have a similar design with a representative sample of the population aged 50 and above (including partners, independent of their age). ELSA started in 2002 and SHARE in 2004 both conduct interviews biannually. SHARE offers information on 29 European countries and Israel, while ELSA covers England. Both surveys conducted life history interviews in their third wave (ELSA 2007, SHARELIFE 2008/09). We did not use the additional life history interview in SHARE wave 7, which included all countries and respondents who joined SHARE after wave three, because information on part-time employment was not available. We used the harmonized life histories for SHARE and ELSA, and the harmonized ELSA data, provided by the Gateway to Global Aging Data (Wahrendorf, Deindl, et al., 2019a, 2019b; Wahrendorf et al. 2023). The harmonized life histories cover five domains: children, partnership, housing, work, and health.

We used a pooled sample of SHARE waves 1 and 2, the two waves before SHARELIFE. For ELSA, we pooled samples from waves 2 and 3 (respondents who participated in the life history interviews) but not from wave 1 because our dependent variable was not consistently available in that wave. Together, these datasets include up to 33,673 respondents in 15 countries: Austria, Germany, Switzerland, France, Belgium, Sweden, Denmark, the Netherlands, Spain, Italy, Greece, the Czech Republic, Poland, Ireland, and England.

Although life histories cover ages 15–80, we used ages 25–50 to ensure that most respondents had completed their education and entered the labor market. The upper age limit was chosen to avoid overlap of respondents’ life histories with care provision measured when they joined SHARE or ELSA. After removing respondents with incomplete life history data (*N* = 1,382), missing information for the dependent variable (*N* = 3,101), and covariates (*N* = 5,571), our analytical sample consisted of 23,619 respondents for care outside the household (10,610 men, 13,009 women) and 20,158 respondents for care inside the household (9,645 men, 10,513 women).

### Dependent variables

We used care information from the pooled SHARE waves 1 and 2 and the pooled ELSA waves 2 and 3. In SHARE, caregiving was defined as providing help with personal care in the previous year to someone living in the same household (in-household caregiving) or to a family member, friend, or neighbor outside the household (out-of-the-household caregiving).

The SHARE question wording was: “*Let us now talk about help within your household. Is there someone living in this household whom you have helped regularly during the last twelve months with personal care, such as washing, getting out of bed, or dressing?*”. Care outside of the household was asked, in SHARE, using the following question: “*In the last twelve months, have you personally given any kind of help listed on this card to a family member from outside the household, a friend or neighbour?*”. The specific types of help were: *personal care* (e.g., dressing, bathing or showering, eating, getting in or out of bed, using the toilet), *practical household help* (e.g., with home repairs, gardening, transportation, shopping, household chores), and *help with paperwork* (such as filling out forms, settling financial or legal matters). Only respondents who engaged with personal care were coded as providing care outside the household. In ELSA respondents were asked “*Did you do any of these activities during the last month?*” with the possible answer: “*Cared for someone*”. A follow-up question provided information on whether respondents lived with the person they cared for, allowing us to determine between care inside and outside the household. This question was only asked only starting from wave 2 onwards. Because SHARE asks only about in-household care among respondents not living alone, we similarly restricted the ELSA sample to respondents not living alone for care within the household.

### Life histories

Wave 3 of the harmonized SHARE and ELSA Gateway file offered eight employment history states: employed full time, employed part-time, self-employed, unemployed, home/family work, retired, full-time education, and other. Respondents who were retired, in full-time education or in the “other” state between age 25 and 50 (between 4 and 8 respondents for each age year) were excluded. The initial harmonized partnership histories included information on whether respondents lived with a partner. We extended this to include whether respondents were widowed, separated, or had other reasons not to live with a partner. For the number of children, we used information on being childless or having one, two, or three or more children from the regular interviews (SHARE waves 1–2; ELSA waves 2–3).

### Independent variables

We used a wide range of control variables likely to impact informal care: socioeconomic conditions, age, household characteristics, availability of siblings and parents, health, and country-level conditions. Some variables may also act as mediators. For example, income may mediate the association between employment histories and informal care. We therefore used a stepwise approach, estimating bivariate regression models between life histories and informal care and multivariate models including all control variables to reduce the risk of adjustment. All variables were taken from SHARE waves 1–2 and ELSA waves 2–3.

Education in SHARE is based on the ISCED 1997 classification. We distinguished low (ISCED 0–2), medium (ISCED 3–4), and high (ISCED 5–6) education. For ELSA, we used the harmonized education variable and distinguished between less than secondary, upper secondary and vocational training, and tertiary education. Income was measured as equivalent household income and divided into country-specific deciles, allowing it to be used as a quasi-metric variable in regression models. Wealth (assets minus debts) was also divided into country-specific deciles. Personal, household, and family conditions included respondent age, household size, whether at least one parent was alive, and whether siblings were alive. Health variables included depression and self-rated health. In ELSA, depression was measured using the CES-D scale; in SHARE, it was measured using the Euro-D scale (Andresen et al. 1994; Prince et al. 1999). We z-standardized both scales to improve comparability. Self-rated health was transformed from the original five-point scale into a dummy variable, distinguishing bad/very bad health from fair or better health.

Country conditions were captured using the framework developed by van Damme et al. (2025). In our sample, The Netherlands, Denmark, and Sweden were classified as strong defamilialization/strong supported familialism; Austria, Germany, Spain, Ireland, England, France, Switzerland, and Belgium as moderate defamilialization/moderate supported familialism; and the Czech Republic, Poland, Italy, and Greece were considered as having familialism-by-default (summarized in Table 1).

**Table 1:** Sample description

	Men		Women	
	Mean/%	SD	Mean/%	SD
**Education**				
Low	42.84		52.94	
Medium	38.96		32.72	
High	18.20		14.34	
**Wealth** (decentiles)	5.97	2.76	5.54	2.85
**Income** (decentiles)	5.92	2.84	5.43	2.84
**HH-size**	2.36	1.03	2.14	1.05
**Age**	64.06	9.10	64.09	9.66
**Parents**				
Both deceased	73.79		73.26	
One alive	20.11		20.39	
Both alive	6.10		6.35	
**Siblings** alive	82.90		82.75	
**Depression** (z-scores)	− 0.29	0.80	0.12	1.00
**Bad health**	24,21		29.38	
**Countries**				
***Strong Df/Sf***	17.66		17.23	
Sweden	5.19		4.97	
Denmark	5.80		5.77	
Netherlands	6.67		6.50	
***Medium Df/Sf***	55.88		54.49	
Austria	2.74		3.36	
Germany	5,52		5.71	
Spain	6.31		6.23	
Ireland	2.05		2.35	
England	19.80		16.77	
France	6.45		7.00	
Switzerland	3.72		4.04	
Belgium	9.28		9.04	
***Familialism-by-default***	26.46		28.27	
Italy	7.73		7.70	
Greece	8.71		9.06	
Czech Republic	5.70		6.49	
Poland	4.32		5.02	
**Sample size**	10,610		13,009	

*Source*: Pooled SHARE Waves 1 and 2 and ELSA Wave 2, Df = defamilialization, Sf = supported familialism

### Method

We applied sequence analysis to summarize employment and partnership histories and applied optimal matching (OM) to quantify the distances between each pair of employment and partner sequences, which are then compared. Sequence analysis with optimal matching originates in biology, specifically in DNA comparisons (Abbott and Tsay 2000). Distance is defined as how much one individual’s sequence must be changed to become identical to another individual’s sequence. The algorithm calculates distances between sequences by counting the number of substitutions or insertions and deletions (indel) needed to make sequences similar. Fewer variations in sequences typically yield fewer possible clusters, whereas greater variations can lead to potentially more clusters. We set substitution costs to 1 and indel costs to 0.5, a common practice (Abbott and Tsay 2000).

We used the resulting distance matrix to group sequences into clusters using Ward’s linkage, which minimizes within-cluster (residual) variance. Thus, similar sequences are grouped together. For men and women, we compared solutions with 2–5 clusters for employment and partnership based on quality measures, cluster sizes, and content validity. We report model fit indices in Appendix 6, together with information on content validity and the size of the smallest cluster. For instance, a three-cluster solution for men’s employment histories produced a very small third cluster (only 36 respondents) without improving the overall model fit. We evaluated three criteria to select the final solution: whether an additional cluster improved model fit, whether it represented a substantively meaningful new group (i.e., captured a distinct aspect), and whether cluster sizes were sufficiently large. Results of the cluster analysis are shown in indexplots, where each line represents one respondent, and his/her states over time. A change in color indicates transitions between states. In Fig. 3, for example, a change from “blue” to “red” indicates a change from “full-time employment” to “unemployment” (see also Vanhoutte et al. (2018) for a short introduction to sequence analysis).

We used the TraMineR, WeightedCluster, and Cluster packages in R for the sequence and cluster analysis (Gabadinho et al. 2011; Ritschard and Studer 2018; Studer 2013). The resulting clusters were then used in stepwise logistic regression models to assess their relation with informal care. We estimated employment histories, partnership, and children separately and then estimated a full model including all control variables and country indicators. We conducted all analyses separately for men and women.

## Results

### Informal care

**Fig. 1:**
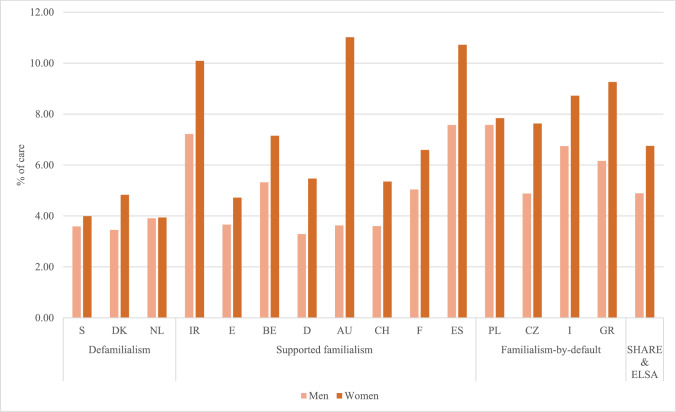
Frequency of informal care inside the household for men and women. *Source*: Pooled SHARE Waves 1 and 2 and ELSA Wave 2, n (men) = 9645, n (women) = 10513

**Fig. 2:**
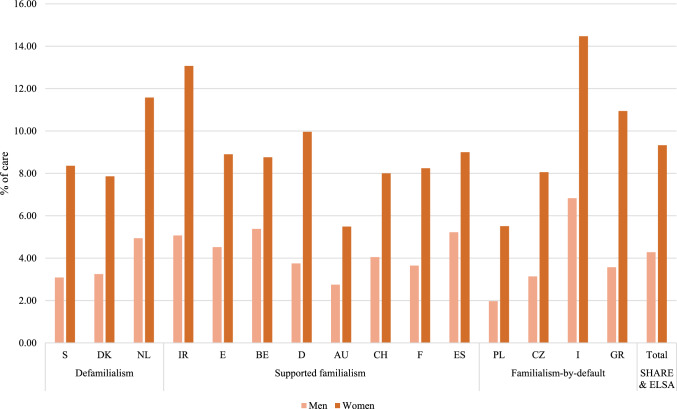
Frequency of informal care outside the household for men and women. *Source*: Pooled SHARE Waves 1 and 2 and ELSA Wave 2, n (men) = 10610, n (women) = 13009

Figures 1 & 2 show the frequency of informal care inside and outside the household among men and women aged 50+ across countries. Around five percent of men provide care inside the household, compared with around seven percent of women. Care outside the household is reported by four percent of men and over nine percent of women. These gender differences are clearly visible across all countries. The rather low caregiving rates observed in SHARE and ELSA are in line with other studies (Brandt et al. 2023). Grouping countries by the Van Damme et al. (2025), long-term care typology reveals no clear care pattern in caregiving prevalence. However, in Sweden, Denmark, and the Netherlands, informal care inside the household is somewhat less prevalent than in the other countries, and the differences between men and women are less pronounced.

### Employment and family histories

**Fig. 3:**
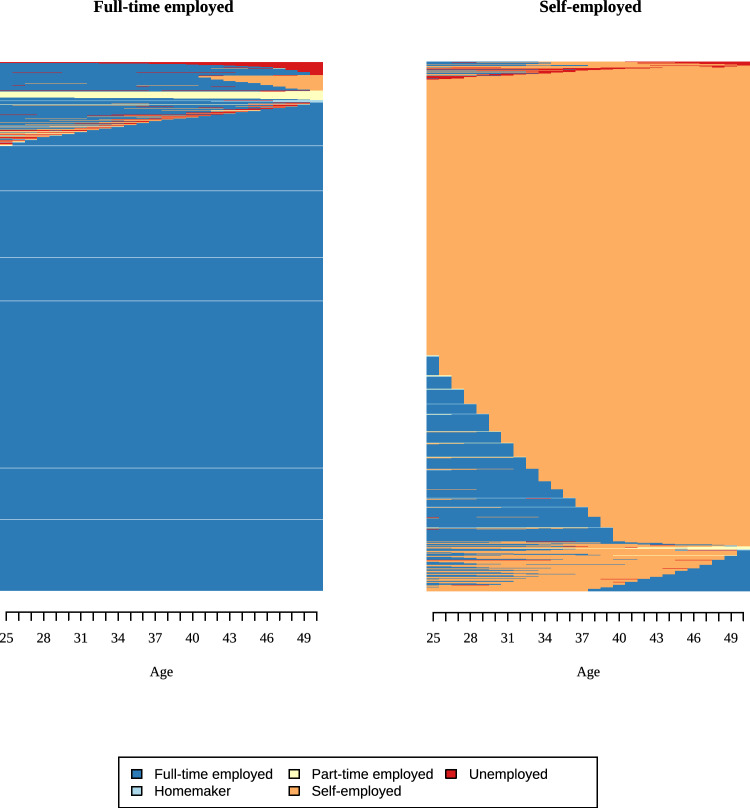
Indexplots of employment clusters for men. Employment: Full-time employed = 8′744; Self-employed = 1′866. *Source*: modified version of life histories of SHARE and ELSA as provided by the Gateway to Global Aging Data platform

**Fig. 4:**
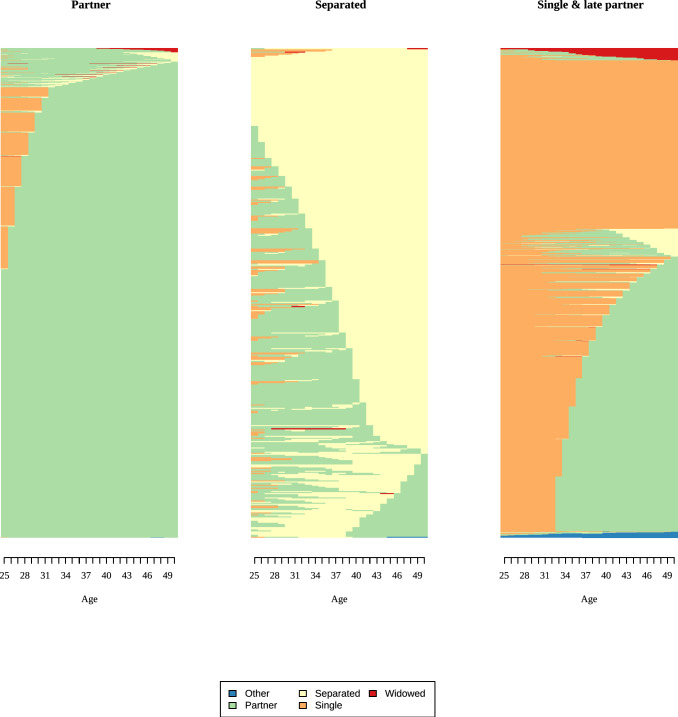
Indexplots of partnership clusters for men. Partnership: Partner = 8842; Separated = 460; Single = 1308. *Source*: Modified version of life histories of SHARE and ELSA as provided by the Gateway to Global Aging Data platform

**Fig. 5:**
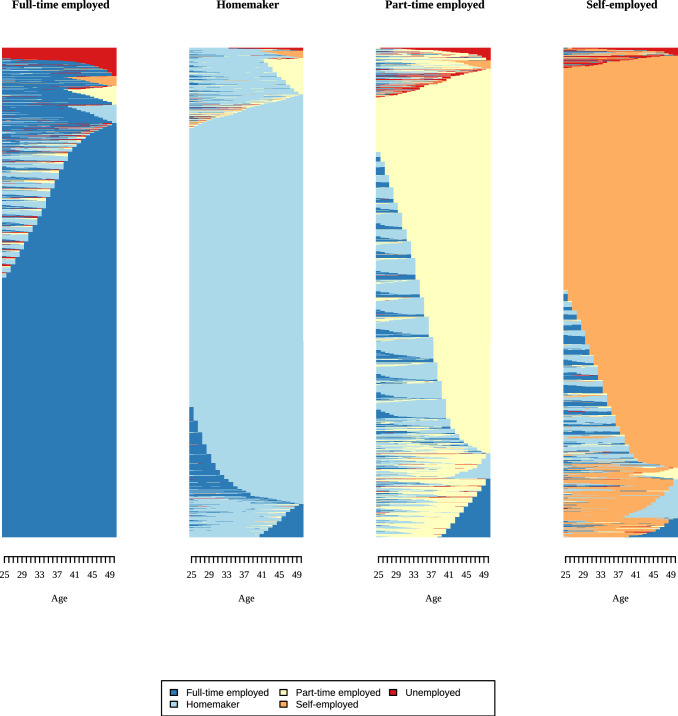
Indexplots of employment clusters for women. Employment: Full-time employed = 5160; Home = 4926; Part-time employed = 1972; Self-employed = 1052. *Source*: modified version of life histories of SHARE and ELSA as provided by the Gateway to Global Aging Data platform

**Fig. 6:**
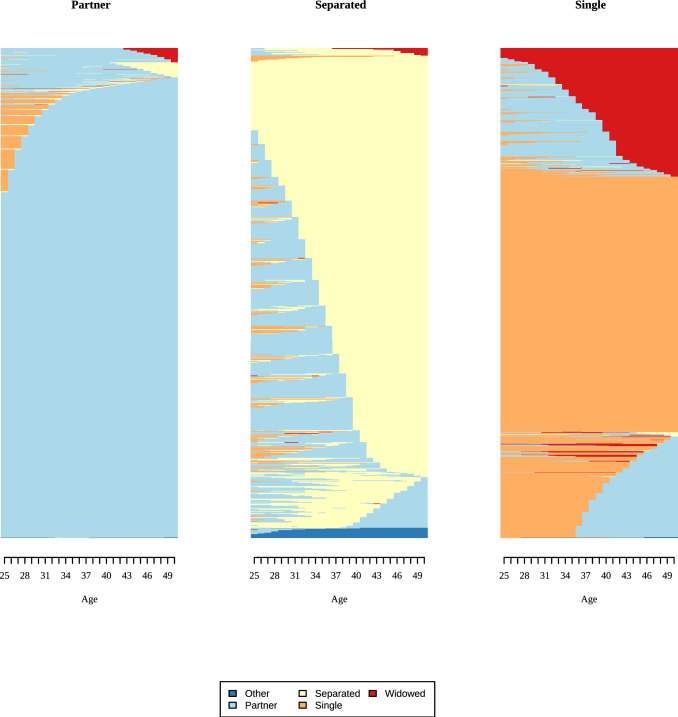
Indexplots of partnership clusters for women. Partnership: Partner = 11,233; Separated = 744; Single = 1033. *Source*: modified version of life histories of SHARE and ELSA as provided by the Gateway to Global Aging Data platform

Employment and family histories differ substantially between men and women (Figs. 3, 4, 5, 6, Tables 2, 3). For employment histories, men and women differed in both the number of clusters and the frequency of each cluster. Based on model fit and content validity, we used two clusters for men: full-time employed and self-employed. Most men were full-time employed, with one-fifth being self-employed. The full-time cluster for men is really consistent, with most men in this cluster employed full time throughout the 25 years observed. At the top of the graph, a small fraction deviates from this pattern, with episodes of unemployment (red) or working part-time (yellow-green). Among the self-employed, some men transitioned from full-time employment to self-employment between the ages of 26 and 40. At the bottom of the graph, a subgroup moved from full time to self-employed, and some even changed back. We identified four distinct clusters among women: full-time employed (40%), staying at home (38%), part-time employment (14%), and self-employed (8%). Female employment histories were more diverse than those of men. Whereas for men, only different forms of employment mattered, a substantial number of women (at the top of the graph) in the full-time employed group changed from different states (homemaker, unemployed, etc.) to being full-time employed. This is even more the case for women in the part-time cluster, where most women changed from homemaker to part-time employment, and some show multiple changes (bottom of third column, Fig. 5).

**Table 2:** Description of employment, partnership, and fertility history for women

	n	Col. %	Cluster name	Dominant state^a^	Mean number of spells
*Employment*					
1	5160	39.66	Full-time employed	Full time (21.98)	6.46
2	4926	37.86	Home	Home (23.35)	5.42
3	1872	14.39	Part-time employed	Part-time (17.02)	8.57
4	1052	8.09	Self-employed	Self-employed (21.36)	6.52
*Partnership*					
1	11233	86.34	Partner	Partner (24.83)	1.37
2	744	5.72	Separated	Separated (17.43)	2.43
3	1033	7.94	Single	Single (17.11)	1.61
*Children*					
Childless	1241	9.54			
1 child	2412	18.54			
2 children	5047	38.79			
3+children	4309	33.12			

*Source*: modified version of life histories of SHARE and ELSA as provided by the Gateway to Global Aging Data platform. ^a^Cells show the most dominant state and the mean number of years spent in it between ages 25 and 50

Differences between men and women were less distinct for partnership histories. Most SHARE and ELSA respondents had a partner for most of the time between ages 25 and 50 (86% women, 83% men). The single cluster revealed some gender differences: About half of the men in this cluster were single between 25 and 50, while the other half partnered in their thirties. Women in the single cluster were single most of the time, with a subset who had a partner but became widows. Compared with employment histories, partnership histories were more stable, with fewer state changes for both men and women. Across all countries, 10% of respondents were childless, around 18% had one child, and about 40% had two children. Slightly more than 30% of respondents had three or more children (Table 3).

**Table 3:** Description of employment, partnership, and fertility history for men

	n	Col. %	Cluster Name	Dominant state^a^	Mean number of spells
*Employment*					
1	8744	82.41	Full-time employed	Full time (24.86)	5.52
2	1866	17.59	Self-employed	Self-employed (22.33)	6.83
*Partnership*					
1	8842	83.34	Partner	Partner (24.51)	1.55
2	460	4.34	Separated	Separated (16.98)	2.58
3	1308	12.33	Single	Single (16.19)	1.80
*Children*					
Childless	1053	9.92			
1 child	1843	17.37			
2 children	4305	40.57			
3+children	3409	32.13			

*Source*: modified version of life histories of SHARE and ELSA as provided by the Gateway to Global Aging Data platform. ^a^Cells show the most dominant state and the mean number of years spent in it between ages 25 and 50

### Life histories and informal care

Table 4 shows the results of stepwise logistic regression models for men. We did not find any significant associations between employment histories and informal caregiving. For partnership histories, we found that, compared to men with a partner, men in the separated cluster were less likely to care for someone inside the household (coef: -0.71, s.e.: 0.36) and more likely to care for someone outside the household (coef: 0.48, s.e.:0.19). Compared to men with 2+children, childless men were also more likely to provide care for someone inside the household (coef: 0.36, s.e.: 0.16), whereas men with one child were more likely to care outside the household (coef: 0.25, s.e.: 0.12).

**Table 4:** Logistic regression of life histories on care inside and outside the household for men

	Inside				Outside			
Variables	Work	Partner	Children	+Controls	Work	Partner	Children	Controls
*Employment histories*								
Full time	Ref			Ref	Ref			Ref
Self-employed	0.13			0.07	0.03			0.02
	(0.12)			(0.12)	(0.12)			(0.13)
*Partner histories*								
Partner		Ref		Ref		Ref		Ref
Separated		− 0.71^**^		− 0.63^*^		0.48^**^		0.38^*^
		(0.36)		(0.37)		(0.19)		(0.20)
Single		0.09		− 0.20		− 0.23		− 0.18
		(0.15)		(0.16)		(0.16)		(0.17)
*Children*								
No children			0.36^**^	0.56^***^			0.08	0.14
			(0.16)	(0.17)			(0.16)	(0.18)
One child			0.13	0.21^*^			0.25^**^	0.22^*^
			(0.12)	(0.13)			(0.12)	(0.12)
Two + children			Ref	Ref			Ref	Ref
*Country*								
Strong Df/Sf				− 0.06				-0.21
				(0.15)				(0.14)
Medium Df/Sf				Ref				Ref
Familialism-by-default				0.24^**^				-0.01
				(0.11)				(0.12)
N	9645				10610			

*Source*: modified version of life histories of SHARE and ELSA as provided by the Gateway to Global Aging Data platform and pooled SHARE Waves 1 and 2 and ELSA Wave 2. ^*^p < 0.1; ^**^p < 0.05; ^***^p < 0.01, Df = defamilialization, Sf = supported familialism, logistic regression coefficient, standard error in parentheses; controls: education, wealth, income, household size, age, parents alive, siblings, depression, health

The results for women were quite different (Table 5). Compared to women who work full time, housewives were more likely to provide care inside the household (coef: 0.21, s.e.: 0.09) but less likely to provide care outside the household (coef: -0.20, s.e.: 0.07). Self-employed women were also more likely to be in-household caregivers (coef: 0.49, s.e.: 0.13). There was also a suggestion that part-time work was negatively associated with in-household caregiving (coef: -0.22, s.e.: 0.13). Women’s partnership histories were not associated with caregiving. Being childless was positively associated with both in-household (coef: 0.24, s.e.: 0.14) and out-of-household (coef: 0.33, s.e.: 0.11) caregiving.

**Table 5:** Logistic regression of life histories on care inside and outside the household for women

	Inside				Outside			
Variables	Work	Partner	Children	+Controls	Work	Partner	Children	+SES
**Employment histories**								
Full time	Ref			Ref	Ref			Ref
Home	0.21^**^			0.03	− 0.20^***^			0.07
	(0.09)			(0.10)	(0.07)			(0.08)
Part-time	− 0.22^*^			− 0.10	0.08			0.12
	(0.13)			(0.14)	(0.09)			(0.10)
Self-employed	0.49^***^			0.31^**^	− 0.07			0.08
	(0.13)			(0.14)	(0.12)			(0.12)
**Partner histories**								
Partner		Ref		Ref		Ref		Ref
Separated		− 0.23		− 0.11		0.04		− 0.17
		(0.21)		(0.22)		(0.13)		(0.14)
Single/widowed		0.03		− 0.18		-0.19		− 0.19
		(0.17)		(0.18)		(0.12)		(0.13)
**Children**								
No children			0.24^*^	0.35^**^			0.14	0.33^***^
			(0.14)	(0.15)			(0.10)	(0.11)
One child			-0.03	0.03			− 0.09	− 0.10
			(0.10)	(0.11)			(0.08)	(0.08)
Two children			Ref	Ref			Ref	Ref
**Country**								
Strong Df/Sf				− 0.33^**^				0.00
				(0.13)				(0.09)
Medium Df/Sf				Ref				Ref
Familialism-by-default				0.12				0.26^***^
				(0.09)				(0.07)
N	10513				13009			

*Source*: modified version of life histories of SHARE and ELSA as provided by the Gateway to Global Aging Data platform and pooled SHARE Waves 1 and 2 and ELSA Wave 2. ^*^p < 0.1; ^**^p < 0.05; ^***^p < 0.01, Df = defamilialization, Sf = supported familialism, logistic regression coefficient, standard error in parentheses; controls: education, wealth, income, household size, age, parents alive, siblings, depression, health

### Country

In the full-model (+controls), countries, grouped by their policies on care in old age, were added to the model. The country coefficients show the correlation between country characteristics and informal care independent of employment, partnership, and children history. Men were more likely to care for a person inside of their household if they lived in a country characterized by *familialism-by-default*. Women living in countries characterized by *strong defamilialism* were less likely to provide care for someone inside the household compared to those living in a country with medium *defamilialism*, while those living in countries characterized by *familialism-by-default* were more likely to provide care outside the household compared to those living in a country with medium *defamilialism*. We additionally calculated interaction effects between care regimes and the life histories, but none of the coefficients was significant at the conventional 5% threshold.

## Discussion

This study is among the first to integrate life history data into caregiving research. Using life history data from SHARE and ELSA, we linked retrospective employment, partnership, and children history (from ages 25 to 50) to caregiving at older ages (50+). Overall, we found that women were more likely than men to provide care, particularly outside the household, with smaller gender differences for in-household caregiving, in line with recent research (Kaschowitz and Brandt 2017; Quashie et al. 2022). As expected in our second hypothesis, we also found that employment histories relate to the likelihood of care provision in later life for women but not for men, while partnership histories matter for men and not for women, with histories of having children influencing men’s and women’s care provision in later life in a similar way.

Women in more family-oriented employment clusters (homemakers) were more likely to provide informal care inside the household, but astonishingly, less likely to provide care to someone outside the household, which contradicts our first hypothesis. This correlation may be linked to partnership status, as the division of labor typically follows the male-breadwinner model (Shelton and John 1996). Therefore, being a housewife for most of their employment history is likely to indicate that these women have a partner and children, making care inside the household more likely. Additionally, self-employed women were more likely to take on care responsibilities. We assume that the greater flexibility associated with self-employment makes it easier for women to combine work with care responsibilities inside the household. While most studies found a direct correlation between working hours and care work (Carrino et al. 2022; Lilly et al. 2007), we did not find this pattern for working part-time over the life course. On the contrary, women who have worked part-time in their adulthood are less likely to take on care responsibilities later in life. The interpretation that the observed correlation between employment and care is largely driven by partnership status is supported by the fact that these correlations are no longer significant after controlling for all covariates (especially partner and household variables).

Partnership histories matter only for men. Men who were separated were less involved in care inside the household and more involved in care outside the household than those with or without a partner. Since having someone to care for is an important condition to become a caregiver (Broese van Groenou and Boer 2016), and most in-household care is for partners (Agree and Glaser 2009), having no partner reduces the likelihood of caregiving. For married men, care is often performed by their wives. Therefore, separated men lack support for their care obligations and are more likely to become caregivers themselves (Haberkern and Szydlik 2010; Schmid et al. 2012). The same mechanism holds for children. Childless men and women are more likely to be caregivers in later life since they have no children to help with their caregiving responsibilities.

At the country level, we found that long-term care policies (measured by country clusters) affect the likelihood that men and women provide care. Using the classification by Van Damme et al. (2025), we found that women were less likely to be in-household caregivers in countries with strong defamilialization/strong supported familialism than in countries characterized by familialism-by-default. Men were also more likely to provide care in countries characterized by familialism-by-default. These findings align with the crowding out-crowding in framework: Countries characterized by strong defamilialization and strong supported familialism crowd out demanding family support such as care provision, while countries which rely on family care make it more necessary that even men assume caregiving responsibilities (Brandt 2013; Brandt et al. 2009; Deindl and Brandt 2017). However, we find no evidence of interactions between care regimes and life histories (results are not shown). This was somewhat surprising, as we had expected (in our fourth hypothesis) life histories to differ across countries, with more gender-equality-oriented welfare states also supporting more gender-equal life histories for women. Future studies should use macro-level indicators like Brandt et al. (2009) or compare selected countries to better understand how employment and family histories interact with caregiving in later life across different contexts.

Our results also showed the importance of care location (i.e., inside and outside the household). As formulated in our third hypothesis, care inside the household is often tied to family-oriented life histories, as it typically involves co-residing family members such as spouses or elderly parents (Kaschowitz and Brandt 2017; Quashie et al. 2022). Partner availability and traditional division of labor—particularly in male-breadwinner family arrangements—may play a significant role in shaping these outcomes. In contrast, caregiving outside the household is differently influenced by family dynamics and may depend on external factors, such as community resources, social networks, and the availability of public support. Overall, our results show that people take on care responsibilities if they have someone to care for (a partner), and need to give care (no partner, no children to share care responsibilities) (Broese van Groenou and Boer 2016). The results also reflect well-established gender differences: Women are more likely to provide care and take on more intensive forms of caregiving (Schmid et al. 2012). For men, the strongest predictor is partner availability—both as a care recipient and as someone who may provide care. For women, the strongest predictors are labor market attachment (and associated flexibility) and being childless.

Our results differ somewhat from McMunn et al. (2020a, b), the only other study with a similar approach to ours. They found that marriage matters for both men and women, whereas in our paper, partnership histories only mattered for men. Additionally, we found that employment was only important for women, particularly being a homemaker or being self-employed. We assume that the differences between both studies are based on the different datasets (European countries vs England), the different time span of the life histories, the older sample in SHARE and ELSA (mean age of 64 vs 54 years for McMunn et al. (2020a, b)), and the consideration of care inside and outside the household, instead of care for parents.

Our study has some limitations. Retrospective interviews are susceptible to recall bias. However, various studies have shown that this problem is rather small. A paper by Wahrendorf et al. (2019a, b, c) was able to demonstrate strong convergence between administrative and life history data. Additional studies based on SHARE and other datasets also revealed high accuracy of life history data (Berney and Blane 1997; Bühler et al. 2022; Havari and Mazzonna 2015). In sequence analysis, the validity of clusters should always be critically discussed. We ran several analyses, in which we coded our variables differently and obtained similar clusters and results in the regression models. One caveat of combining sequence analysis with cluster analysis is that differences in timing and sequences are not always fully displayed. For example, in our “single” cluster, men who remained single and men who found a partner later in life were grouped together. Therefore, these differences in timing and sequences are “lost” in our analysis.

Our empirical analysis did not analyze indirect effects between life histories and caregiving. Structural equation models, for example, would allow differentiation between direct and indirect effects over the life course and further investigation of the impact of life course events on caregiving in later life. Future studies should pursue this line of research, which was outside of the scope of this paper.

We also did not differentiate between the recipients of care, but only between care inside and outside the household. Most care is directed at close family, typically parents or spouses. Some studies find that caregiver without a partner and children were most likely to care for their parents (Leopold et al. 2014), other studies don’t find similar relationships (Haberkern et al. 2013). Therefore, a careful analysis of care recipients might refine our results.

The strength of our paper is that we have examined the impact of previous life conditions (employment and family histories) on informal caregiving among older people in Europe. However, our respondents were interviewed in the early 2000s. Since then, female labor force participation and divorce rates among older people have increased (Glaser et al. 2024), and men’s life courses are becoming more complex (McMunn et al. 2015), with more men working part-time and taking leave to care for their children. Therefore, the roles of employment and family histories are likely to become more significant and to have a substantial impact on both the availability and provision of informal care in the future.

## Data Availability

The ELSA data are available via the UK Data Service (SN 5050). SHARE data are available at https://share-eric.eu/

## Appendix

See Tables 6, 7, 8, 9, 10.

**Table 6:** Cluster solution of employment and partnership clusters

Number of clusters	Mean within distances	Mean between distances	WB-Ratio	PBC	ASW	Obs. in smallest cluster
*Employment*						
Men						
2	9.30	17.75	0.52	0.60	0.46	237
3	8.26	18.01	0.46	0.71	0.50	36
4	7.55	16.83	0.45	0.66	0.39	36
Women						
2	13.2	18.9	0.70	0.48	0.29	1339
3	11.4	18.4	0.62	0.56	0.32	917
4	9.87	18.5	0.53	0.65	0.37	318
5	9.28	17.9	0.52	0.61	0.32	315
*Partnership*						
Men						
2	8.94	15	0.60	0.56	0.39	297
3	7.87	14.4	0.55	0.62	0.39	160
4	7.28	14.2	0.51	0.65	0.34	116
*Women*						
2	9.94	15.6	0.64	0.47	0.35	340
3	8.33	15.8	0.53	0.65	0.41	147
4	7.66	14.8	0.52	0.58	0.33	132

*Source*: modified version of life histories of SHARE and ELSA as provided by the Gateway to Global Aging Data platform. PBC: WB-Ratio: Within/between cluster distance ratio; PBC: Point Biserial Correlation; and ASW: Average Silhouette

**Table 7:** Logistic regression of life histories on care inside the household for men (complete table)

	Work	Partner	Children	Life histories	+ SES	+ Household	+ Health	+ Countries
*Employment histories*								
Full time								
Self-employed	0.13			0.14	0.17	0.10	0.10	0.07
	(0.12)			(0.12)	(0.12)	(0.12)	(0.12)	(0.12)
*Partner histories*								
Partner								
Separated		− 0.71^**^		− 0.75^**^	− 0.80^**^	− 0.63^*^	− 0.66^*^	− 0.63^*^
		(0.36)		(0.36)	(0.36)	(0.36)	(0.36)	(0.37)
Single		0.09		− 0.02	− 0.06	− 0.17	− 0.18	− 0.20
		(0.15)		(0.16)	(0.16)	(0.16)	(0.16)	(0.16)
*Children*								
No children			0.36^**^	0.39^**^	0.37^**^	0.53^***^	0.54^***^	0.56^***^
			(0.16)	(0.17)	(0.17)	(0.17)	(0.17)	(0.17)
One child			0.13	0.14	0.15	0.21*	0.20	0.21^*^
			(0.12)	(0.12)	(0.12)	(0.13)	(0.13)	(0.13)
Two and more children								
*Control variables*								
**Education**								
Less than secondary								
Upper secondary					-0.17	-0.07	-0.05	-0.02
					(0.11)	(0.11)	(0.11)	(0.11)
Tertiary					-0.35^**^	-0.27^*^	-0.24	-0.19
					(0.15)	(0.16)	(0.16)	(0.16)
**Wealth**					-0.09^***^	-0.09^***^	-0.09^***^	-0.08^***^
					(0.02)	(0.02)	(0.02)	(0.02)
**Income**					-0.00	0.03	0.03	0.03
					(0.02)	(0.02)	(0.02)	(0.02)
**Household size**						0.28^***^	0.28^***^	0.25^***^
						(0.04)	(0.05)	(0.05)
**Age**						0.04^***^	0.04^***^	0.04^***^
						(0.01)	(0.01)	(0.01)
**Parents alive**								
Both deceased								
One alive						0.26^*^	0.27^*^	0.27^*^
						(0.14)	(0.14)	(0.14)
Both alive						0.37	0.38^*^	0.39^*^
						(0.23)	(0.23)	(0.23)
**Siblings**						-0.13	-0.13	-0.12
						(0.12)	(0.12)	(0.12)
**Depression**							0.14^**^	0.15^**^
							(0.06)	(0.06)
Health							0.14	0.11
							(0.11)	(0.11)
**Countries**								
Strong Df/Sf								-0.06
								(0.15)
Medium Df/Sf								Ref
Familialism-by-default								0.24^**^
								(0.11)
Constant	-2.99^***^	-2.96^***^	-3.02^***^	-3.03^***^	-2.40^***^	-6.16^***^	-6.09^***^	-6.10^***^
	(0.05)	(0.05)	(0.06)	(0.06)	(0.14)	(0.55)	(0.55)	(0.55)
*N*	9645	﻿9645	﻿9645	﻿9645	﻿9645	﻿9645	﻿9645	

^*^*p* < 0.1; ^**^*p* < 0.05; ^***^*p* < 0.01. Coefficients, t-value in parentheses, *Source*: modified version of life histories of SHARE and ELSA as provided by the Gateway to Global Aging Data platform

**Table 8:** Logistic regression of life histories on care outside the household for men (complete table)

	Work	Partner	Children	Life histories	+ SES	+ Household	+ Health	+ Countries
*Employment histories*								
Full time								
Self-employed	0.03			0.06	0.05	0.03	0.02	0.02
	(0.12)			(0.13)	(0.13)	(0.13)	(0.13)	(0.13)
*Partner histories*								
Partner								
Separated		0.48^**^		0.45^**^	0.52^***^	0.37^*^	0.37^*^	0.38^*^
		(0.19)		(0.19)	(0.20)	(0.20)	(0.20)	(0.20)
Single		− 0.23		− 0.27	− 0.21	− 0.19	− 0.19	− 0.18
		(0.16)		(0.17)	(0.17)	(0.17)	(0.17)	(0.17)
*Children*								
No children			0.08	0.16	0.18	0.16	0.16	0.14
			(0.16)	(0.17)	(0.17)	(0.18)	(0.18)	(0.18)
One child			0.25^**^	0.25^**^	0.24^**^	0.24^*^	0.24^*^	0.22^*^
			(0.12)	(0.12)	(0.12)	(0.12)	(0.12)	(0.12)
Two and more children								
*Control variables*								
**Education**								
Less than secondary								
Upper secondary					0.25^**^	0.13	0.14	0.13
					(0.11)	(0.11)	(0.11)	(0.12)
Tertiary					0.28^**^	0.17	0.16	0.17
					(0.14)	(0.14)	(0.14)	(0.14)
**Wealth**					0.07^***^	0.07^***^	0.07^***^	0.07^***^
					(0.02)	(0.02)	(0.02)	(0.02)
**Income**					0.02	0.01	0.01	0.01
					(0.02)	(0.02)	(0.02)	(0.02)
**Household size**						-0.03	-0.03	-0.04
						(0.05)	(0.05)	(0.05)
**Age**						-0.01	-0.01	-0.01
						(0.01)	(0.01)	(0.01)
**Parents alive**								
Both deceased								
One alive						0.77^***^	0.77^***^	0.77^***^
						(0.12)	(0.12)	(0.12)
Both alive						0.73^***^	0.73^***^	0.72^***^
						(0.18)	(0.18)	(0.18)
**Siblings**						-0.02	-0.02	-0.01
						(0.13)	(0.13)	(0.13)
**Depression**							0.10	0.10*
							(0.06)	(0.06)
Health							-0.10	-0.13
							(0.13)	(0.13)
**Countries**								
Strong Df/Sf								-0.21
								(0.14)
Medium Df/Sf								
Familialism-by-default								-0.01
								(0.12)
Constant	-3.11^***^	-3.11^***^	-3.16^***^	-3.18^***^	-3.94^***^	-3.27^***^	-3.25^***^	-3.14^***^
	(0.05)	(0.05)	(0.06)	(0.06)	(0.16)	(0.60)	(0.60)	(0.60)
*N*	10610	﻿10610	﻿10610	﻿10610	﻿10610	﻿10610	﻿10610	﻿10610

^*^*p* < 0.1; ^**^*p* < 0.05; ^***^*p* < 0.01. Coefficients, t-value in parentheses, *Source*: modified version of life histories of SHARE and ELSA as provided by the Gateway to Global Aging Data platform

**Table 9:** Logistic regression of life histories on care inside the household for women (complete table)

	Work	Partner	Children	Life histories	+ SES	+ Household	+ Health	+ Countries
*Employment histories*								
Full time								
Home	0.21^**^			0.23^**^	0.08	0.02	0.01	0.03
	(0.09)			(0.09)	(0.09)	(0.10)	(0.10)	(0.10)
Part-time	− 0.22^*^			− 0.21	− 0.24^*^	− 0.19	− 0.17	− 0.10
	(0.13)			(0.13)	(0.13)	(0.13)	(0.13)	(0.14)
Self-employed	0.49^***^			0.50^***^	0.43^***^	0.31^**^	0.32^**^	0.31^**^
	(0.13)			(0.13)	(0.14)	(0.14)	(0.14)	(0.14)
*Partner histories*								
Partner								
Separated		− 0.23		− 0.18	− 0.27	− 0.11	− 0.15	− 0.11
		(0.21)		(0.21)	(0.21)	(0.22)	(0.22)	(0.22)
Single/widowed		0.03		− 0.05	− 0.14	− 0.22	− 0.19	− 0.18
		(0.17)		(0.18)	(0.18)	(0.18)	(0.18)	(0.18)
*Children*								
No children			0.24^*^	0.30^**^	0.29^**^	0.38^**^	0.37^**^	0.35^**^
			(0.14)	(0.15)	(0.15)	(0.15)	(0.15)	(0.15)
One child			− 0.03	0.00	0.01	0.06	0.05	0.03
			(0.10)	(0.10)	(0.10)	(0.11)	(0.11)	(0.11)
Two and more children								
*Control variables*								
**Education**								
Less than secondary								
Upper secondary					− 0.28^***^	− 0.18^*^	− 0.13	− 0.12
					(0.09)	(0.10)	(0.10)	(0.10)
Tertiary					− 0.33^**^	− 0.24^*^	− 0.17	− 0.11
					(0.14)	(0.14)	(0.14)	(0.15)
**Wealth**					− 0.05^***^	− 0.05^***^	− 0.04^***^	− 0.04^**^
					(0.01)	(0.02)	(0.02)	(0.02)
**Income**					− 0.04^***^	− 0.02	− 0.02	− 0.02
					(0.01)	(0.02)	(0.02)	(0.02)
**Household size**						0.23^***^	0.22^***^	0.20^***^
						(0.04)	(0.04)	(0.04)
**Age**						0.03^***^	0.03^***^	0.03^***^
						(0.01)	(0.01)	(0.01)
**Parents alive**								
Both deceased								
One alive						0.04	0.03	0.03
						(0.11)	(0.11)	(0.11)
Both alive						0.15	0.16	0.17
						(0.18)	(0.18)	(0.18)
**Siblings**						0.21^*^	0.21^*^	0.23^**^
						(0.11)	(0.11)	(0.12)
**Depression**							0.23^***^	0.23^***^
							(0.04)	(0.04)
Health							0.16^*^	0.13
							(0.09)	(0.09)
**Countries**								
Strong Df/Sf								
								-0.33^**^
Medium Df/Sf								(0.13)
Familialism-by-default								Ref
								0.12
								(0.09)
Constant	− 2.73^***^	− 2.62^***^	− 2.64^***^	− 2.75^***^	− 2.03^***^	− 4.88^***^	− 4.90^***^	− 4.89^***^
	(0.07)	(0.04)	(0.05)	(0.08)	(0.13)	(0.43)	(0.43)	(0.44)
*N*	10513	10513	10513	10513	10513	10513	10513	10513

^*^*p* < 0.1; ^**^*p* < 0.05; ^***^*p* < 0.01. Coefficients, t-value in parentheses, *Source*: modified version of life histories of SHARE and ELSA as provided by the Gateway to Global Aging Data platform.

**Table 10:** Logistic regression of life histories on care outside the household for women (complete table)

	Work	Partner	Children	Life histories	+ SES	+ Household	+ Health	+ Countries
*Employment histories*								
Full time								
Home	-0.20^***^			-0.20^***^	-0.06	0.05	0.04	0.07
	(0.07)			(0.07)	(0.07)	(0.08)	(0.08)	(0.08)
Part-time	0.08			0.07	0.09	0.05	0.03	0.12
	(0.09)			(0.09)	(0.09)	(0.09)	(0.09)	(0.10)
Self-employed	-0.07			-0.08	-0.00	0.06	0.07	0.08
	(0.12)			(0.12)	(0.12)	(0.12)	(0.12)	(0.12)
*Partner histories*								
Partner								
Separated		0.04		-0.02	0.04	-0.17	-0.19	-0.17
		(0.13)		(0.13)	(0.13)	(0.14)	(0.14)	(0.14)
Single/widowed		-0.19		-0.30^**^	-0.24^*^	-0.18	-0.18	-0.19
		(0.12)		(0.13)	(0.13)	(0.13)	(0.13)	(0.13)
*Children*								
No children			0.14	0.20^*^	0.22^**^	0.32^***^	0.31^***^	0.33^***^
			(0.10)	(0.11)	(0.11)	(0.11)	(0.11)	(0.11)
One child			-0.09	-0.11	-0.11	-0.10	-0.10	-0.10
			(0.08)	(0.08)	(0.08)	(0.08)	(0.08)	(0.08)
Two and more children								
*Control variables*								
**Education**								
Less than secondary								
Upper secondary					0.32^***^	0.13^*^	0.13^*^	0.15^**^
					(0.07)	(0.07)	(0.07)	(0.07)
Tertiary					0.39^***^	0.17^*^	0.16^*^	0.22^**^
					(0.09)	(0.09)	(0.09)	(0.10)
**Wealth**					0.05^***^	0.04^***^	0.04^***^	0.04^***^
					(0.01)	(0.01)	(0.01)	(0.01)
**Income**					0.02^**^	0.01	0.01	0.01
					(0.01)	(0.01)	(0.01)	(0.01)
**Household size**						-0.06^*^	-0.05	-0.07^**^
						(0.03)	(0.03)	(0.03)
**Age**						-0.03^***^	-0.03^***^	-0.03^***^
						(0.00)	(0.00)	(0.00)
**Parents alive**								
Both deceased								
One alive						0.86^***^	0.85^***^	0.86^***^
						(0.07)	(0.07)	(0.07)
Both alive						0.47^***^	0.46^***^	0.47^***^
						(0.12)	(0.12)	(0.12)
**Siblings**						0.10	0.09	0.09
						(0.09)	(0.09)	(0.09)
**Depression**							0.17^***^	0.18^***^
							(0.03)	(0.03)
Health							-0.24^***^	-0.27^***^
							(0.08)	(0.08)
**Countries**								
Strong Df/Sf								0.00
								(0.09)
Medium Df/Sf								Ref
Familialism-by-default								0.26^***^
								(0.07)
Constant	-2.21^***^	-2.26^***^	-2.27^***^	-2.18^***^	-2.87^***^	-0.74^*^	-0.76^**^	-0.90^**^
	(0.05)	(0.03)	(0.04)	(0.06)	(0.10)	(0.38)	(0.38)	(0.38)
*N*	13009	13009	13009	13009	13009	13009	13009	13009

^*^*p* < 0.1; ^**^*p* < 0.05; ^***^*p* < 0.01. Coefficients, t-value in parentheses, *Source*: modified version of life histories of SHARE and ELSA as provided by the Gateway to Global Aging Data platform.
